# Noise-Induced Hearing Loss in Gerbil: Round Window Assays of Synapse Loss

**DOI:** 10.3389/fncel.2021.699978

**Published:** 2021-07-27

**Authors:** Penelope W. C. Jeffers, Jérôme Bourien, Artem Diuba, Jean-Luc Puel, Sharon G. Kujawa

**Affiliations:** ^1^Program in Speech and Hearing Bioscience and Technology, Harvard University, Boston, MA, United States; ^2^Eaton-Peabody Laboratories, Massachusetts Eye and Ear, Boston, MA, United States; ^3^Institute for Neurosciences of Montpellier, University of Montpellier, INSERM, Montpellier, France; ^4^Department of Otolaryngology-Head and Neck Surgery, Harvard Medical School, Boston, MA, United States

**Keywords:** auditory nerve fiber, cochlear synaptopathy, cochlear neural degeneration, hair cell, noise-induced hearing loss, peri-stimulus time response, sensorineural hearing loss, spontaneous rate

## Abstract

Previous work in animals with recovered hearing thresholds but permanent inner hair cell synapse loss after noise have suggested initial vulnerability of low spontaneous rate (SR) auditory nerve fibers (ANF). As these fibers have properties of response that facilitate robust sound coding in continuous noise backgrounds, their targeted loss would have important implications for function. To address the issue of relative ANF vulnerabilities after noise, we assessed cochlear physiologic and histologic consequences of temporary threshold shift-producing sound over-exposure in the gerbil, a species with well-characterized distributions of auditory neurons by SR category. The noise exposure targeted a cochlear region with distributed innervation (low-, medium- and high-SR neurons). It produced moderate elevations in outer hair cell-based distortion-product otoacoustic emission and whole nerve compound action potential thresholds in this region, with accompanying reductions in suprathreshold response amplitudes, quantified at 24 h. These parameters of response recovered well with post-exposure time. Chronic synapse loss was maximum in the frequency region initially targeted by the noise. Cochlear round window recorded mass potentials (spontaneous neural noise and sound-driven peri-stimulus time responses, PSTR) reflected parameters of the loss not detected by the conventional assays. Spontaneous activity was acutely reduced. Steady-state (PSTR plateau) activity was correlated with synapse loss in frequency regions with high concentrations of low-SR neurons, whereas the PSTR onset peak and spontaneous round window noise, both dominated by high-SR fiber activity, were relatively unaltered across frequency in chronic ears. Together, results suggest that acute targets of noise were of mixed SR subtypes, but chronic targets were predominantly low-SR neurons. PSTRs captured key properties of the auditory nerve response and vulnerability to injury that should yield important diagnostic information in hearing loss etiologies producing cochlear synaptic and neural loss.

## Introduction

Sound coding by the cochlea must accurately represent the very large spans of level and frequency information present in the original sound stimulus. It must do so while preserving temporal information and by extracting these details in non-ideal coding environments, for example in a signal degraded by noise or other distortions. This is supported through the activation of populations of auditory nerve fibers (ANFs) that are distinguished by different spontaneous rates (SR) of firing and demonstrate different thresholds and dynamic ranges of rate coding responses. These ANFs, through their direct and highly specialized synaptic communications with the sensory inner hair cells (IHCs) of the cochlea, comprise the vast majority of fibers responsible for carrying this information toward the brain.

Over the last decade, a number of investigations have provided evidence that the synapses between IHCs and ANFs that provide for this information flow are the most vulnerable elements in several etiologies of sensorineural hearing loss (SNHL) common in humans, including noise and aging. These studies showed that many synapses disappear acutely after noise, even for exposures that cause only transient threshold shifts and no hair cell loss (Kujawa and Liberman, 2009; Lin et al., 2011; Fernandez et al., 2015). They revealed, in aging, that declines occur gradually over the lifespan, beginning before either outer hair cell (OHC) loss or the resultant cochlear threshold shifts first appear (Sergeyenko et al., 2013; Parthasarathy and Kujawa, 2018). Loss of synapses renders associated ANFs silent.

Initially studied in mouse models, similar observations have now been made in multiple mammalian species (Kujawa and Liberman, 2015 for review). In humans, studies of age-graded archival temporal bones have revealed that loss of ANFs outstrips IHC loss by a factor of two in “normal aging” cases (Wu et al., 2019) and that acoustic injury accelerates this age-related primary neural degeneration (Wu et al., 2021). Although assessment of human temporal bone histopathology can provide direct documentation of the deafferentation, assessment of possible consequences to human auditory function remains in early stages. Thus, given the fundamental role of ANFs in sound coding, understanding properties of ANF responses in health, and the relative vulnerabilities of ANF fiber subtypes to injury, is key to studying auditory deficits.

Initial studies hypothesized that a subset of ANFs was chronically most vulnerable to noise, and, based on subtotal losses and post-exposure recovery of thresholds, low-SR neurons were suggested as possible initial targets (for review, Liberman and Kujawa, 2017). Neurophysiological study supported this hypothesis; at a steady-state time after recovery from noise-induced threshold shifts, low-SR ANFs appeared reduced in cochlear regions tonotopically appropriate to the noise insult. In contrast, high-SR fibers were well represented, with sensitive thresholds and well-preserved properties of tuning and suprathreshold response (Furman et al., 2013). These high-SR neurons are largely responsible for coding lowest-level signals in quiet, whereas low-SR neurons, which maintain robust responses in continuous noise backgrounds (Costalupes et al., 1984), are suggested to be the key to recognition of complex signals in noise. Such an outcome might help explain speech-in-noise difficulties that are commonly reported, even in individuals with normal thresholds (Bharadwaj et al., 2014), and may help explain performance differences in ears with similar audiometric thresholds.

Low-SR neuron activity, however, is difficult to extract from the ‘whole-nerve’ responses typically captured to assess auditory nerve function in experimental and clinical settings. By virtue of their generally elevated thresholds (Liberman, 1978), delayed first spike onset (Rhode and Smith, 1985; Versnel et al., 1990), and broad distribution (jitter) of first spike latencies (Oliver et al., 2006; Buran et al., 2010; Bourien et al., 2014; Huet et al., 2016), low-SR neuron loss can be particularly well hidden from detection by conventional assessments of hearing and of neural function, creating significant diagnostic challenges (Huet et al., 2019).

Recent work has suggested a promising approach to clarifying neural targets of noise, using a round-window recorded peri-stimulus time response (PSTR) of ANFs to sound (Batrel et al., 2017). The PSTR resembles the classically recorded PST histogram of a single ANF response, with its prominent onset response (peak) decaying exponentially to a lower steady-state value (plateau). The PSTR does not estimate directly the proportion of low- vs. high-SR based fiber loss; however, in simultaneous recordings of single ANF PST histograms and round-window PSTRs, the PSTR peak-to-plateau ratio correlated positively with the mean SR of the fibers tuned to the PSTR probe frequency (Huet et al., 2021). Consequently, a targeted loss of low-SR fibers after noise may elevate the mean SR of surviving fibers, inducing a potential change on the PSTR peak and/or plateau. In complement to such sound-driven responses, the neural “noise” that can be detected from the round window in the absence of acoustic stimulation (Dolan et al., 1990; Cazals and Huang, 1996; McMahon and Patuzzi, 2002) is characterized by a broad spectral peak centered near 0.8–1.0 kHz. Because the amplitude of this peak reflects the overall spontaneous firing of the ANFs, which is dominated by high-SR fibers, it may provide an indirect estimate of high-SR fiber survival (Batrel et al., 2017).

Here, we established a gerbil model of temporary noise-induced threshold shift with cochlear synaptopathy. In gerbil, the SR-based distribution of ANFs is well characterized. It varies as a function of cochlear location and displays a higher proportion of low-SR fibers than in mouse (Schmiedt, 1989; Ohlemiller and Echteler, 1990; Taberner and Liberman, 2005; Bourien et al., 2014; Petitpré et al., 2020). This specificity makes the gerbil a useful model in which to study different SR-based pools of ANFs within the same cochlea for their vulnerability to noise. We recorded distortion product otoacoustic emissions (DPOAE) to evaluate the functional integrity of the OHCs and compound action potentials (CAP) of the auditory nerve to assess ANF firing synchrony at the onset of the acoustic stimulation. Our electrophysiological approach also included the recording of ensemble ANF spontaneous activity at the level of the cochlear round window (e.g., Dolan et al., 1990) and sound-evoked PSTRs to assess their sensitivity to synapse loss and their ability to reflect the relative vulnerability of low-vs. high-SR fibers to noise.

## Materials and Methods

### Animals and Groups

Mongolian gerbils (*Meriones unguiculatus*) of both sexes were used for all experiments. Animals were born and housed in a colony from breeders obtained from Charles River Laboratories. At age 14 weeks (wk; ±5%) gerbils were noise-exposed and assigned to groups to be tested at various post-exposure times (24 h, 2 wk, or 4 wk after noise). Age-matched, unexposed animals otherwise held identically served as controls. All procedures were approved by the Institutional Animal Care and Use Committee of the Massachusetts Eye and Ear.

### Noise Exposure

Awake gerbils were placed, singly and unrestrained, in a small wire mesh cage suspended directly below the acoustic horn of a sound delivery loudspeaker that extended into a reverberant exposure chamber. A one-octave band of noise (2.8–5.6 kHz) was delivered at 100 dB SPL for 2 h. Calibration to the target level was accomplished immediately preceding each exposure session. Sound levels at different locations within the holding cage varied within 1 dB of the target level.

### Physiology

Physiologic testing was conducted in an acoustically and electrically shielded chamber heated to 34°C. Gerbils were anesthetized with ketamine (100 mg/kg ip) and xylazine (5 mg/kg ip). Anesthesia was maintained with periodic administration of ketamine (33–50 mg/kg ip). Heart rate, temperature, and oxygen saturation were monitored throughout testing. A National Instruments PXI-based system with 24-bit digital input/output boards generated all stimuli and captured all responses, controlled by custom LabVIEW-based software (details at: https://masseyeandear.org/research/otolaryngology/eaton-peabody-laboratories/engineering-core). Signals were delivered using a custom, closed acoustic assembly comprising two miniature sound delivery speakers (CDMG15008-03A, CUI) and a detection microphone (FG-23329-PO7) to measure sound pressure in the ear canal. Responses were amplified (10,000×; Grass P511) with a 10–3,000 Hz (CAP) or 3–10,000 Hz (PSTR) pass band. The left ear of each animal was tested.

#### DPOAE

Distortion product otoacoustic emissions were elicited with stimuli consisting of two pure tones (f1 and f2) presented at frequencies defined by f2/f1 = 1.2 and at levels defined by L1 = L2 + 10 dB. Captured from ear canal pressure measurements, DPOAEs of the frequency 2f1-f2 were recorded as functions of increasing stimulus level (L2 = 0–80 dB SPL, 5 dB steps) at 10 f2 frequencies from 2 to 44 kHz. From the growth functions, iso-response curves were interpolated to determine DPOAE thresholds, defined as the minimum level required to elicit a DPOAE of −5 dB SPL.

#### CAP

Compound action potentials of the auditory nerve were recorded using a wire recording electrode (platinum-iridium) placed at the round window niche, with subdermal needle electrodes at the vertex (reference) and tail base (ground). CAPs were elicited by tone pips (0.5 ms rise-fall, 5 ms plateau, 16/s). Stimulus frequencies matched DPOAE f2 values, and the level was increased in 5 dB steps from below threshold to 90 dB SPL. Opposite-polarity stimulus pairs (128 tone pips/polarity) were presented for each frequency-level combination. Responses were amplified (10,000×), filtered (10–3,000 Hz), and averaged. Offline, peaks corresponding to N1 and P1 of the action potential were identified visually from stacked waveforms, aided by custom software. The threshold was defined as the lowest level at which repeatable response peaks were evident, and peak-to-peak values of the N1-P1 components were used to calculate response amplitudes.

#### Round Window Noise

Electrical activity from the round window in the unstimulated condition (e.g., round window “noise”) was recorded with the same electrode used for CAP assessment. The detected activity was captured over 40 s, amplified (10,000×), and its overall power spectral density (PSD) was estimated using Welch’s method (*pwelch* function using MATLAB language, 2,048 samples per segment, 0% overlapped, rectangular window, sampling rate 100,000 samples/s). The coordinate (frequency, *x*-axis; amplitude, *y*-axis) of the spectral peak occurring in the 900 Hz range of the PSD was detected using the *max* MATLAB function (search window 300–1,200 Hz). The amplitude of the 900-Hz component in the round window noise was also estimated by applying a bandpass filter (300–1,200 Hz, 2nd-order Butterworth filter) to the 40-s trace and calculating the overall root-mean-square (RMS) level. Spectral peak amplitude, frequency, and RMS level were compared across groups of noise-exposed animals and controls.

#### PSTRs

Peri-stimulus time responses were elicited using 1/3 octave band noise bursts (trapezoidal envelope, 200 ms duration, 1 ms rise/fall) with center frequencies at each of the 10 CAP test frequencies, levels from 0 to 80 dB SPL in 10 dB steps, and 50 presentations per frequency-level combination. Each “presentation” comprised a pair of bursts presented in opposite polarities to minimize the hair cell-based cochlear microphonic. The seed of the pseudorandom noise generation was refreshed at the first burst of each pair to ensure independence of the stimulus waveform across presentations. Half sums from each presentation pair were filtered (300–1,200 Hz) and the temporal envelope extracted by full-wave rectification and smoothing (1-ms time span). PSTRs were then obtained by averaging the resulting signals as in Huet et al. (2021). The onset-peak amplitude of the PSTR was estimated using the *max* MATLAB function, during the first 6 ms of the response. The plateau amplitude of the PSTR was measured by averaging the PSTR samples during the last 50 ms of the response.

### Immunostaining of Cochlear Whole Mounts

Immediately following the testing, subsets of animals from each group were transcardially perfused with 4% paraformaldehyde in 0.1 M phosphate buffer, followed by intralabyrinthine perfusion of fixative through the oval and round windows. Cochleae were post fixed for 2 h at room temperature and decalcified in 0.12 M EDTA for 72 h. The left (tested) cochlea was processed for these studies.

The organ of Corti was microdissected into nine pieces, transferred to a sucrose solution (30% sucrose in PBS), permeabilized by freeze/thawing, and blocked in 5% normal horse serum with 0.3% Triton X-100 in PBS for 1 h. Pieces were incubated for ~20 h at 37°C with primary antibodies then incubated for 2 h at 37°C with secondary antibodies. All antibodies were diluted in 1% normal horse serum with 0.3% Triton X-100 in PBS. IHC bodies were labeled with an antibody against myosin VIIa, a component of hair cell stereocilia and cytoplasm (rabbit anti-myosin VIIa, Proteus Biosciences, 1:200; AlexaFluor 647 donkey anti-rabbit, 1:200). Presynaptic ribbons were labeled with an antibody against a predominant ribbon component, C-terminal binding protein 2 (mouse IgG1 anti-CtBP2, BD Biosciences, 1:200; AF 568 goat anti-mouse IgG1, 1:1,000). Post-synaptic glutamate patches were labeled with an antibody against the GluR2 subunit of AMPA-selective glutamate receptors (mouse IgG2a anti-GluA2, Millipore, 1:2,000; Alexa Fluor 488 goat anti-mouse IgG2a, 1:1,000). Cochlear segments were mounted in Vectashield (Vector Laboratories) on a glass microscope slide, arranged from apex to base.

### Hair Cell and Synapse Quantification

Immunostained cochlear segments were imaged at low power (Leica DM5500 epifluorescence microscope, 10× air objective, N.A. 0.4) for quantification of inner and outer hair cell loss. A cochlear frequency map was produced from the same images for each organ of Corti using a custom plug-in for Image J, based on the place-frequency map for gerbil (Müller, 1996). For synapse quantification, confocal *z*-stacks were acquired (Leica TCS SP5) using a glycerol-immersion objective (63×, N.A. 1.3) and 3.17× digital zoom. The x-y dimensions were fixed for all stacks at 1,024 × 512 pixels. The y dimension included and extended slightly beyond the tectorial-to-basilar membrane length of IHCs. The z dimension was selected manually for each stack to capture the full modiolar-to-pillar extent of every IHC in the *x*-*y* frame. Approximately 15–17 IHCs were imaged at each frequency location by acquiring two adjacent *z* stacks (0.33 μm spacing).

Image stacks were imported to Amira (ThermoFisher Scientific) to quantify hair cells, pre-synaptic ribbons, and post-synaptic glutamate receptor patches. IHCs were inspected for overall morphology based on their myosin-stained cell bodies and quantified based on their CtBP2-stained nuclei. In Amira, a 3D representation of each stack was produced and rotated during quantification to avoid undercounting ribbons obstructed by each other at certain viewing angles. Synapses were quantified as paired pre-synaptic ribbon/post-synaptic glutamate receptor patch puncta at seven cochlear locations from 0.5 to 32 kHz.

## Results

### Noise-Induced DPOAE and CAP Threshold Shifts Were Similar and Reversible

**Figure 1:**
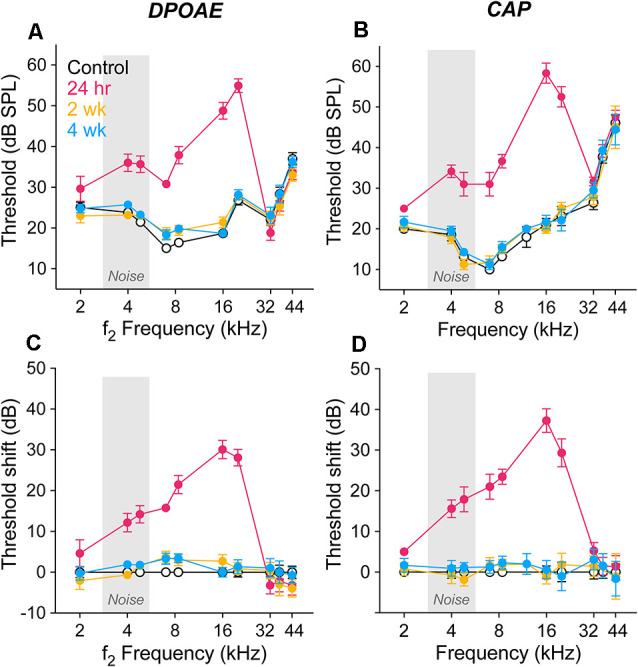
Response thresholds and shifts from control. Thresholds of DPOAEs **(A)** and CAPs **(B)** are shown as response means ± SEM for unexposed controls (white/black) and groups of gerbils held 24 h (magenta), 2 wk (orange), and 4 wk (blue) after noise (2.8–5.6 kHz, 100 dB SPL, 2 h). Corresponding shifts from control values are shown in panels **(C)** and **(D**). The exposure noise band is indicated in light gray. Group sizes were: control (14), 24 h (7), 2 wk (7), 4 wk (9). For some group means, error bars do not exceed symbol boundaries. The color scheme applies to all figures. ANOVA models were run at 8 and 16 kHz using hierarchical tests at each frequency in a predefined sequence. Control vs. exposed groups was significant for both DPOAE and N1-P1 at both 8 and 16 kHz (*p* < 0.001 in all four comparisons). 24 h vs. 2 and 4 wk groups was significant by both measures and at both frequencies (*p* < 0.001 in all four comparisons). DPOAE and CAP thresholds at 2 wk and 4 wk post-exposure were not different at either frequency (*p* = 0.80 and *p* = 0.45 at 8 kHz and 16 kHz, respectively). For all threshold analyses, alpha = 0.05.

Threshold sensitivity was assessed in groups of gerbils held 24 h, 2 wk or 4 wk after exposure (2.8–5.6 kHz, 100 dB SPL, 2 h) and in age-matched animals held identically except for the single exposure. Our aim was to produce robust, but reversible, threshold elevations as characterized previously (Kujawa and Liberman, 2009; Lin et al., 2011). In Figure 1, the effects of the exposure on DPOAEs and CAPs are shown in threshold audiograms (Figures 1A,B) and as shifts from control values (Figures 1C,D). Twenty-four hours after exposure, threshold shifts were maximum at 16 kHz for both DPOAEs and CAPs (30.1 ± 2.3 dB and 37.3 ± 2.9 dB, respectively) and fell rapidly below 4 kHz and above 20 kHz, consistent with the parameters of the overexposure stimulus and known nonlinearities in the cochlear response to sound (e.g., Robles and Ruggero, 2001; Kujawa, 2016 for review). We ran ANOVA models at two frequencies within this range, 8 and 16 kHz, where hierarchical tests showed a significant effect of exposure (control vs. exposed groups) in both DPOAE and CAP response thresholds at both frequencies (*p* < 0.001 for all comparisons). Thresholds recovered significantly with post-exposure time: comparisons of the 24 h group with the 2 and 4 wk groups, by both measures and at both 8 and 16 kHz, were significant (*p* < 0.001 for all). DPOAE and CAP thresholds at 2 wk and 4 wk post-exposure were not different at either frequency. The comparable magnitude of threshold shift in the two response types is consistent with OHC involvement in the acute injury.

### Response Amplitudes Revealed Different Patterns of Recovery After Noise

**Figure 2:**
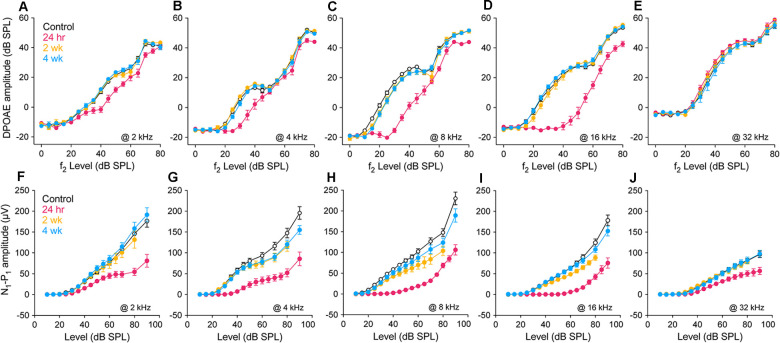
Suprathreshold response amplitudes. Amplitude vs. stimulus level functions for DPOAEs (L2 = 0–80 dB SPL) and CAPs (10–90 dB SPL) in unexposed and exposed gerbils 24 h, 2 wk and 4 wk after noise. 2f1-f2 DPOAE **(A–E)** and N1-P1 of the CAP **(F–J)** at 2, 4, 8, 16, and 32 kHz. All data points are group means ± SEM; group sizes are as given in the Figure 1 caption. Statistical tests were performed for amplitude data at 4, 8 and 16 kHz, at 30 dB SL to facilitate comparison with peri-stimulus time response (PSTR) responses shown in Figure 6. Control vs. exposed groups (alpha = 0.05): DPOAE amplitudes differed at 8 kHz (*p* < 0.001) and 16 kHz (*p* < 0.001) but not 4 kHz (*p* = 0.07); N1-P1 amplitudes were reduced in exposed groups at all three frequencies (*p* < 0.001). 24 h vs. 2 wk, 4 wk (alpha = 0.025): DPOAE amplitudes were significantly different at 8 and 16 kHz (*p* < 0.001 for both) but not at 4 kHz (*p* = 0.37); N1-P1 amplitudes recovered significantly with post-exposure time at 4, 8, and 16 kHz (*p* < 0. 001 at each frequency). Control vs. 2 wk, 4 wk (alpha = 0.025): DPOAE amplitudes of the exposed groups were less than control at 8 kHz (*p* = 0.018) but not at 4 or 16 kHz (*p* = 0.20 and *p* = 0.64, respectively); similarly, neural responses of the exposed groups were less than control at 8 kHz (*p* = 0.02) but not at 4 or 16 kHz (*p* = 0.16 and *p* = 0.28, respectively). Two week and 4 wk amplitudes were not different at 4, 8, or 16 kHz in the DPOAE (*p* = 0.65, *p* = 0.91, and *p* = 0.54) or the N1-P1: (*p* = 0.86, *p* = 0.25, and *p* = 0.07) responses.

Response amplitudes, as functions of increasing stimulus level, were recorded for DPOAEs and CAPs at all threshold test frequencies. Figure 2 displays results for five frequencies ranging from below (2 kHz; Figures 2A–F) to above (32 kHz; Figures 2E–J) the region of temporary threshold shift at each of the three post-exposure times, as compared to controls. Consistent with the pattern of injury revealed by the threshold measures at 24 h post-exposure, DPOAE growth functions were shifted to the right and linearized at 4, 8, and 16 kHz (Figures 2B–D), most dramatically at the frequency of maximum threshold shift, 16 kHz (Figure 2D). In contrast, response alterations were small for frequencies outside this region (Figures 2A–E). CAP response amplitudes at 24 h were largely reduced from control at all frequencies shown (Figures 2F–J). Effects of noise were assessed at 30 dB SL for CAPs, to allow comparison with PSTR metrics, and in DPOAEs, for consistency. Statistical analyses were performed on amplitude data at 4, 8, and 16 kHz. As in threshold analyses (Figure 1), hierarchical testing began by considering all four groups for an effect of exposure.

In these comparisons, control DPOAE amplitudes differed significantly from the exposed groups at 8 kHz (*p* < 0.001) and 16 kHz (*p* < 0.001) but not 4kHz (*p* = 0.07). Similarly, amplitudes of the 24 h group were significantly different from the longer-held groups for f2 frequencies of 8 and 16 kHz (*p* < 0.001 for both) but not 4 kHz (*p* = 0.37). When these longer-held groups were compared to control, there was a difference in DPOAE amplitudes at 8 kHz (*p* = 0.018) but not at 4 or 16 kHz (*p* = 0.20 and *p* = 0.64, respectively). Across all three frequencies, N1-P1 amplitudes of the control group were significantly different from those of the exposed groups (*p* < 0.001). Amplitudes of the longer-held groups (2 wk and 4 wk) differed from the 24 h group at all frequencies (*p* < 0. 001 at each frequency) and from the control group at 8 kHz (*p* = 0.02) but not at 4 or 16 kHz (*p* = 0.16 and *p* = 0.28, respectively). At all tested frequencies, amplitudes of the 2 and 4 wk groups were not significantly different by either metric.

### Synapse Loss Persisted in Region of Maximum Acute Noise Injury

**Figure 3:**
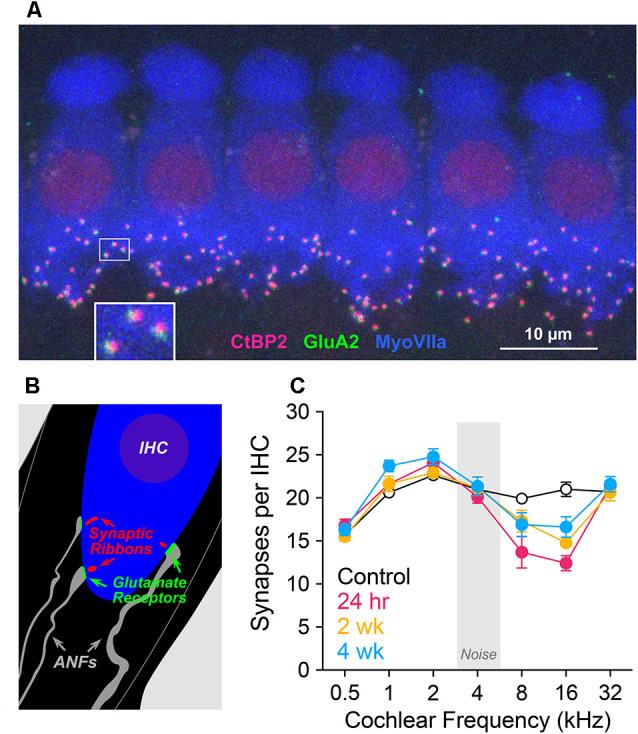
Synapse counts. **(A)** Confocal maximum projection of six adjacent inner hair cells (IHCs) in the 2 kHz cochlear location, immunolabeled for presynaptic ribbons (CtBP2, red), postsynaptic receptor patches (GluA2, green), and IHCs (myosin VIIa, blue). At the base of one hair cell, a small white rectangle indicates the region displayed below at higher magnification. **(B)** Schematic of the IHC area showing presynaptic and postsynaptic elements. **(C)** Synapses per IHC at cochlear locations corresponding to CAP test frequencies, plotted as group means ± SEM in unexposed and exposed gerbils 24 h, 2 wk, and 4 wk after noise, *n* = 4 animals/group. ANOVA models were run at 8 and 16 kHz using hierarchical tests at each frequency in a predefined sequence. Comparison of control vs. all exposed groups (alpha = 0.05) indicated a significant effect of exposure at both 8 kHz (*p* = 0.02) and 16 kHz (*p* < 0.001). At 8 kHz, 24 h vs. 2 wk, 4 wk (alpha = 0.025) was not significant (*p* = 0.11), nor was control vs. 2 wk, 4 wk (*p* = 0.07). At 16 kHz, 24 h vs. 2 wk, 4 wk (alpha = 0.025) was significant (*p* = 0.005), as was control vs. 2 wk, 4 wk (*p* < 0.001). Pairwise comparison of 2 wk vs. 4 wk was not significant at either frequency.

Structures supporting sensory-to-neural communication include glutamate-releasing presynaptic ribbons of IHCs and postsynaptic glutamate receptor patches of ANF terminals. To quantify these elements, we immunostained these structures, as seen in confocal xy projections (Figure 3A). These immunolabeled IHC-ANF synapses are schematized in Figure 3B and quantified in Figure 3C. Noise effects on IHC synapses (Figure 3C) were concentrated in cochlear regions corresponding to frequencies of maximum TTS. To analyze these effects, ANOVA models were run at 8 and 16 kHz using hierarchical tests at each frequency in a predefined sequence. Synapses per IHC in exposed cochleae were significantly reduced from controls (*p* = 0.02 at 8 kHz; *p* < 0.001 at 16 kHz). At 16 kHz, despite significant recovery with post-exposure time (2 and 4 wk vs. 24 h, *p* = 0.005) values remained reduced compared to control (2 and 4 wk vs. control, *p* = 0.001). A similar pattern is apparent, but not statistically significant, at the 8 kHz cochlear location (*p* = 0.1, *p* = 0.07). Synapse counts at 2 and 4 wk were not different from each other at 8 or 16 kHz. Synapse counts above and below these frequencies appeared unaffected by the band-limited noise at any post-exposure time assessed. We note that synapse counts in control ears at 1 kHz are slightly smaller than lab norms and may account for the apparent elevations from control at 1 kHz in the noise-exposed ears. In the same ears, outer and inner hair cell losses were <5% at all cochlear locations assessed for all groups.

### Spontaneous Round Window-Recorded Neural Activity Was Acutely Reduced After Noise

**Figure 4:**
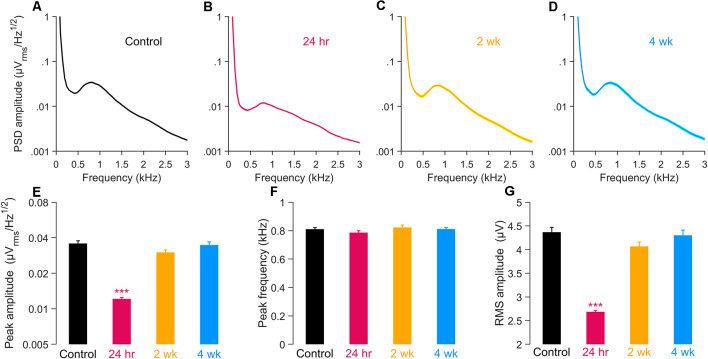
Power spectral density (PSD) of spontaneous round window activity. **(A–D)** PSD in unexposed **(A)** and exposed gerbils 24 h **(B)**, 2 wk **(C)**, and 4 wk **(D)** after noise. **(E,F)** Amplitude **(E)** and frequency **(F)** of the spectral peak in the same groups. **(G)** Root-mean-square (RMS) amplitude of the PSD after applying 300–1200 Hz band-pass filter. In all panels, data are shown as means ± SEM, with group sizes as given in the Figure 1 caption. **(E–G)** Groups were compared using a one-way ANOVA and a *post hoc* test for multiple comparisons of groups; ***p < 0.001.

The electrical activity that can be detected by a round window electrode in the absence of intentional stimulation (round window noise; Dolan et al., 1990; Cazals and Huang, 1996; McMahon and Patuzzi, 2002; Batrel et al., 2017) reflects the unsynchronized, spontaneous discharge of ANFs. This activity can be highlighted by computing the power spectrum density (PSD) of the round window noise. The PSD shows a spectral peak in the vicinity of 900 Hz, as seen in Figures 4A–D. In gerbil, due to the close proximity of the auditory nerve to this recording site (Chamberlain, 1977), the unitary contributions of ANFs to the 900 Hz peak is assumed to be independent of the fiber’s characteristic frequency (i.e., weak base-to-apex gradient of contribution) but significantly dominated by the high-SR fibers (i.e., strong low-to high-SR gradient of contribution).

We considered whether this spontaneous activity was acutely and/or chronically altered by noise exposure by comparing the amplitude and nominal center frequency of this spectral peak activity among noise-exposed groups (at 24 h, 2 wk, or 4 wk; Figures 4B–D) with unexposed controls (Figure 4A). Results quantified at 24 h revealed a dramatic reduction, to about 34% of control, in PSD peak amplitude, without significant change in the frequency location of the spectral peak (Figures 4E,F). By 2 weeks post-exposure, these parameters of the activity had returned to control levels and were largely stable at 4 weeks. Expressed as RMS amplitudes of the 300–1,200 Hz band-pass filtered signal, the same time-dependent relationships hold, with a large decline in RMS amplitude evident at 24 h, followed by recovery to control levels (Figure 4G). Together, results suggest significant declines in the activity of high-SR neurons in the acute post-exposure time frame, with good recovery evident in chronic ears. Such recordings provide little direct information concerning the functional integrity of low-SR ANFs.

### PSTRs Tracked Synapse Loss After Mild Noise Exposure

Peri-stimulus firing adaptation is an important feature of ANF response, largely determined by the IHC-ribbon synapse machinery (Moser and Beutner, 2000; Beutner et al., 2001; Goutman and Glowatzki, 2007). Peri-stimulus time histograms (PSTHs) of single ANF responses to sound (Westerman and Smith, 1984) reveal a peak of activity at the onset of stimulation followed by adaptation to a steady-state firing plateau that persists with continued stimulation. These features of the PSTH are remarkably well-preserved in the global ANF electrical activity accessible at the level of the cochlear round window, the peri-stimulus time response, PSTR.

**Figure 5:**
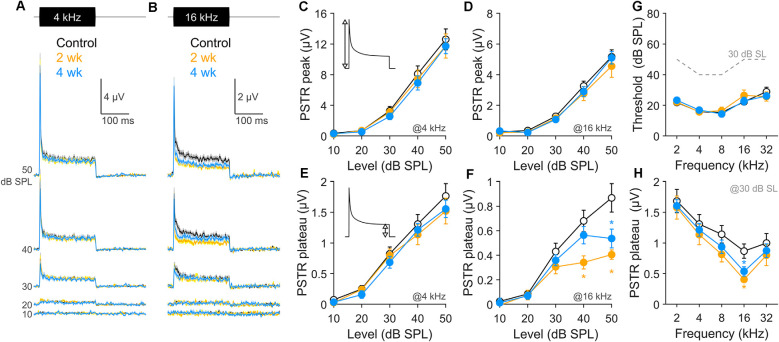
PSTR peak and plateau after noise exposure. **(A,B)** Population PSTRs obtained in response to noise bursts centered at 4 kHz **(A)** and 16 kHz **(B)**, for sound levels 10–50 dB SPL, in 10 dB steps. Group sizes are as listed in the Figure 1 caption. **(C–F)** The amplitude of the PSTR peak at 4 kHz **(C)** and 16 kHz **(D)** and of the PSTR plateau at 4 kHz **(E)** and 16 kHz **(F)** as a function of level. Insets in **(C,E)** indicate the relevant measurements. **(G)** PSTR derived threshold. For reference, the gray dashed line indicates the sound levels at 30 dB above PSTR threshold (i.e., 30 dB SL). **(H)** The amplitude of the PSTR plateau at 30 dB SL as a function of the probe frequency. Data are means ± SEM; *p < 0.05, Student’s *t*-test.

Here, with the same RW electrode used to record the CAP and the spontaneous round window activity, we recorded PSTRs evoked by a train of narrow-band noise bursts centered at the probe frequencies used to elicit the CAP. To avoid an excessive spread of excitation, we adjusted the sound level at 30 dB above the threshold, which has been shown to be sufficient to recruit low-SR fibers in the gerbil (Huet et al., 2016). In Figure 5, we considered several characteristics of the PSTR recorded 2 and 4 wk after moderate noise exposure, as compared to unexposed controls. At these post-exposure times, PSTR thresholds (Figure 5G) and DPOAE and CAP thresholds (Figure 1) were not different from control levels; thus, noise-induced shifts and OHC dysfunction are not confounds to interpretation.

Two frequencies were selected for comparison; 4 kHz, in a cochlear region where no synapse loss was seen, and 16 kHz, where the maximum synapse loss occurred (see Figure 3). In unexposed animals, the onset-sensitive PSTR peaks, shown in Figures 5A,B (black traces) and quantified in Figures 5C,D (black lines), display amplitudes that grew with level and varied by frequency. PSTR peak amplitudes were not persistently altered by the noise either within (16 kHz) or outside (4 kHz) the cochlear region maximally injured by the noise. In contrast, the steady-state plateau of the response showed persistent declines at 16 kHz but not 4 kHz (compare Figures 5E,F), summarized in Figure 5H for stimulation at 30 dB SL. Although outcomes relative to SR subtype vulnerability after noise can be influenced by the expected frequency/cochlear location of the noise injury and OHC involvement at short post-exposure times, effects on the PSTR plateau isolated to the 16 kHz region, in combination with the complete lack of chronic noise effects on PSTR peak responses at either frequency, together suggest persistent dysfunction of low-SR fibers.

It is widely accepted that sound-evoked gross potentials recorded at the round window are dominated by the response of ANFs populating the cochlear region tuned to the probe frequency (Kiang et al., 1965; Ozdamar and Dallos, 1978). Unitary responses at the gerbil round window appear independent of fiber CF (Batrel et al., 2017). However, estimating the number of fibers contributing to gross metrics remains difficult (Bourien et al., 2014). In control gerbils, the number of synapses (fibers) per IHC was well matched at 4 and 16 kHz (20.8 ± 0.3 at 4 kHz vs. 20.9 ± 0.8 at 16 kHz; Figure 3C). However, CAP amplitude differences were large (80.8 ± 6 μV at 4 kHz vs. 45.6 ± 2.9 μV at 16 kHz, measured 30 dB above threshold; Figure 2). Similar differences were observed in the PSTR, with a peak of 12.6 ± 1.3 μV at 4 kHz vs. 5.2 ± 0.4 μV at 16 kHz and plateau of 1.7 ± 0.2 μV at 4 kHz vs. 0.9 ± 0.1 μV at 16 kHz (Figure 5). These differences in CAP and PSTR amplitudes at 4 and 16 kHz may be strongly influenced by frequency-dependent differences in the shapes of their respective neural tuning curves (Schmiedt, 1989; Ohlemiller and Echteler, 1990). Fibers tuned to 16 kHz display narrower tuning curves than those tuned to 4 kHz, especially evident at lower levels of stimulation (i.e., 30 dB above threshold). Sound stimulation at 16 kHz will therefore recruit a more restricted number of fibers sharply tuned to the probe stimulation than 4-kHz stimulation, which will recruit a larger number of fibers and thus display broader tuning. Fibers tuned to 16 kHz also exhibit higher saturation firing rates compared to 4 kHz-tuned fibers (Ohlemiller et al., 1991; Huet et al., 2016); however, this difference is probably not sufficient to counterbalance the tuning curve effect.

To circumvent these inherent frequency-dependent effects, we normalized the CAP and PSTR amplitudes measured in exposed animals relative to values from unexposed animals. Because noise-induced synaptopathy does not change the neural tuning curve and rate- vs.-intensity functions of remaining fibers (Furman et al., 2013), we assume that the decrease of the CAP and PSTR normalized amplitudes can be attributed to synapse loss.

**Figure 6:**
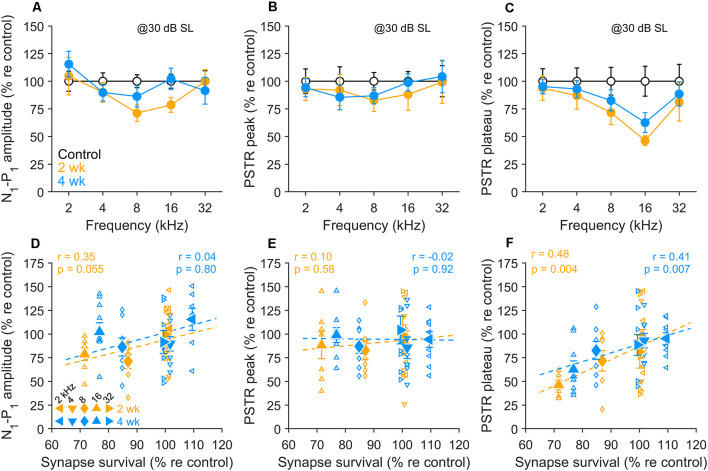
Correlations between electrophysiological indices and synapse survival. **(A–C)** N1-P1 CAP amplitude **(A)**, PSTR peak **(B)**, and PSTR plateau **(C)** in unexposed and exposed gerbils 2 wk and 4 wk after noise. Means (± SEMs) are normalized to unexposed group values. CAP and PSTR measured at 30 dB above PSTR threshold. **(D–F)** Individual data (small symbols), group means (large symbols), and linear fits (dashed lines) for 2- and 4-wk held animals compared to control, for the electrophysiological indices shown in panels **(A–C)** and synapse survival data shown in Figure 3. Means are ± SEM, *n* = 4 animals/group. Correlations were tested using the Pearson correlation coefficient; *r* and *p*-values are given on the individual panels **(D–F)**.

As displayed in Figure 6A, CAP amplitudes in the 8–16 kHz region showed persistent declines at 2 wk post-exposure, with some recovery occurring by 4 wk (see also Figure 2). PSTR peak amplitudes over the same range of frequencies (Figure 6B) showed non-significant changes from control at both post-exposure times. This onset-dominated response was a poor predictor of synapse survival in these ears (Figure 6E). CAP N1-P1 amplitudes (Figure 6D) fared somewhat better, with outcomes that varied with frequency, and a generally moderate predictive value for synapse loss. In comparison, PSTR plateau, representing activity from a population of ANFs distributed across SR groups, including low-SR fibers, showed persistent, ~ 40% declines in tonotopically-appropriate regions (Figure 6C) and correlations with synapse survivals at both 2 and 4 wk after noise (Figure 6F).

**Figure 7:**
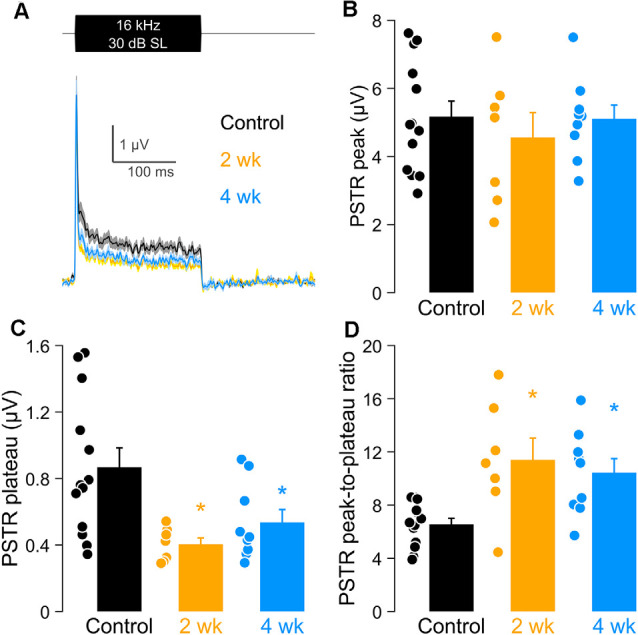
PSTR peak, plateau, and peak-to-plateau ratio after noise exposure at 16 kHz. **(A)** PSTRs in response to a burst of third-octave band noise presented at 30 dB above PSTR threshold. **(B–D)** PSTR peak **(B)**, plateau **(C)**, and peak-to plateau ratio **(D)** in unexposed and exposed animals, with group sizes as given in the Figure 1 caption. Closed circles indicate individual data. **(B–D)** Data are shown as means ± SEM and compared using a one-way ANOVA and a *post hoc* test for multiple comparisons of groups; *p < 0.05.

In Figure 7, we focused on PSTRs obtained at 16 kHz, the region of maximum synaptic loss. Direct comparisons of the PSTR stimulus waveform (Figure 7A), PSTR peak and plateau amplitudes, and their derived peak-to-plateau ratios clearly reveal the sensitivity, particularly of the ratiometric response, in capturing this hidden noise-induced injury (Figures 7B–D). These parameters of the PSTR are plotted for the 2 wk post-exposure, 4 wk post-exposure, and unexposed control groups. Peak PSTR values (Figure 7B) did not differ significantly among the control and noise-exposed groups. In contrast, significantly smaller PSTR plateaus (Figure 7C), driving significantly larger PSTR peak-to-plateau ratios (Figure 7D), were seen for both groups of noise-exposed ears. These results suggest a preferential vulnerability of low-SR neurons which is not well captured by the onset-driven PSTR peak and CAP (see also Figure 6).

## Discussion

### Development of a Gerbil Model of Noise-Induced Cochlear Synaptopathy

Sound overexposure can result in hair cell damage or loss, associated with threshold elevation, degraded frequency tuning, and loss of critical cochlear nonlinearities (Liberman and Kiang, 1978; Schmiedt, 1984). We now know that noise exposure also can lead to extensive cochlear neuronal degeneration. This primary cochlear deafferentation is initially seen as the loss of IHC synapses with primary ANFs.

Synaptic losses after noise can be dramatic; up to ~50% of synapses can disappear within minutes of exposure in tonotopically-relevant cochlear regions (Kujawa and Liberman, 2009; Fernandez et al., 2020), silencing affected fibers. The condition has been labeled “hidden hearing loss,” because substantial synaptic and neural losses can be present in ears with normal or recovered thresholds. Beyond the relative insensitivity of the threshold audiogram to such loss, commonly utilized whole nerve responses like the CAP and ABR wave 1 are not ideally suited to reflect the loss of hypothesized primary targets of noise, the low-SR ANFs (Schmiedt, 1989; Ohlemiller and Echteler, 1990; Bourien et al., 2014; Batrel et al., 2017).

To facilitate the study of this deafferentation and to provide insights regarding ANF vulnerabilities by SR subtype, we established a model of noise-induced cochlear deafferentation in gerbil, a species with well-characterized distributions of auditory neurons by SR subtype and a range of hearing significantly overlapping that of humans (Ryan, 1976). The exposure used in these studies produced acutely moderate elevations in hair cell- and neural-based response thresholds and reduced suprathreshold amplitudes that showed good recovery with post-exposure time. Effects on these responses were maximum for stimulus frequencies corresponding to cochlear regions with a distributed representation of ANFs with low, medium, and high spontaneous rates of firing, at least as observed in unexposed ears. As with other species studied, we observed post-noise synapse loss in the same frequency region demonstrating maximal post-noise neural response declines. For the exposure we tested, chronic losses were small.

### ANF Populations in Normal Ears and Vulnerability to Insult

Sound coding relies on auditory nerve fibers, the majority of which communicate singly with a single IHC, initiating the neural spike trains that carry information toward the brain. These ANFs display spontaneous rates of firing ranging from ~0 to over 100 spikes per second (Kiang et al., 1965). Their driven rates differ by their thresholds of activation and saturation characteristics, supporting intensity coding over a large dynamic range. Auditory nerve fibers comprise three SR-based pools, as described by Liberman (1978) in the cat: high-SR (>18 spikes/s) fibers evidenced the lowest thresholds, medium-SR (0.5–18 spikes/s) fibers had higher thresholds, and the low-SR (<0.5 spikes/s) population had the highest minimum, mean, and maximum thresholds. The dynamic ranges of low-SR fibers are shifted to higher levels by their thresholds and are larger than those of medium- and high-SR fibers (Winter et al., 1990; Taberner and Liberman, 2005).

Distributions of high-, medium- and low-SR fibers remain incompletely described in multiple mammalian species and are virtually uncharacterized in humans. In gerbil, the majority of afferents apical to the 3–4 kHz cochlear location are high-SR fibers, whereas SR subtypes are more evenly distributed basally, where low-SR fibers are concentrated (Schmiedt, 1989; Ohlemiller and Echteler, 1990; Müller, 1996; Bourien et al., 2014; Huet et al., 2016, 2019). Sound-driven response properties of ANFs also demonstrate this transition, yielding different distributions of slopes, saturation rates, and dynamic ranges, as well as different interactions among these response parameters (Ohlemiller et al., 1991). With a roughly 4–5× greater proportion of low-SR fibers in the cochlear base compared to the apex, and the differing regional phenotypes that result, the gerbil provides a powerful means to address questions of ANF vulnerability and regenerative potential.

We have hypothesized that the relative insensitivity of thresholds to cochlear synaptopathy/deafferentation arises because cochlear neurons most vulnerable to noise are those with high thresholds and low spontaneous firing rates (Furman et al., 2013; Kujawa and Liberman, 2015). These low-SR fibers do not contribute to normal thresholds in quiet but are key to coding transient stimuli in continuous background noise that saturates the responses of sensitive high-SR fibers. Such SR-varying responses have led to speculation that low-SR neuropathy is a major contributor to the classic impairment in SNHL, i.e., difficulties with speech discrimination in difficult (noisy) listening environments, which can impair performance with or without threshold elevation.

### Detecting and Characterizing Auditory Neuropathies

Understanding relative vulnerabilities of ANFs to injury is fundamental to clarifying functional consequences of the neural loss. We are faced with challenges, however, in capturing information about low-SR neuron survival and function. Although the CAP of the auditory nerve and wave I of the auditory brainstem response are commonly used to assess the functional integrity of cochlear neurons, these responses best capture the onset responses of ANFs, which are dominated by high-SR fibers. The activity of low-SR neurons, with generally higher thresholds, delayed first spike onset and broad distribution (jitter) of first spike latencies, is not as well reflected in such responses to sound (Bourien et al., 2014).

The global peri-stimulus time response (PSTR) captures key properties of AN response that should yield important diagnostic information in hearing loss etiologies producing IHC synaptic and neural loss (Batrel et al., 2017; Huet et al., 2021). In recent work, simultaneous recordings of single ANF responses and of the round window recorded PSTR showed comparable onset and adaptation kinetics (Huet et al., 2021). The time constant of the PSTR peak decreased with the level of stimulation, whereas short-term kinetics were level-independent. The peak-to-plateau ratio decreased with probe frequency. When compared with SRs of the single fibers, peak-to-plateau ratios reflected the heterogeneity of ANF distribution in gerbil; that is, a majority of high-SR fibers in the apex, and a more balanced distribution at the base (Huet et al., 2021). This modeling and experimental work suggests that the PSTR onset peak is dominated by the synchronous activation of the high-SR fibers, whereas the PSTR plateau reflects more broadly/equally the fiber subtypes. In studies reported here, we show that the PSTR tracks synaptic/neural loss after noise injury, even for relatively small losses, and does so with sensitivity to SR subtype.

### Sensitivity to Low-SR Neuropathy

The recordings of spontaneous activity obtained in control and noise-exposed gerbils in this series suggest acute noise effects on ANFs with high spontaneous rates of firing. In gerbil, the cochlear portion of the auditory nerve runs in close proximity to the round window (Sokolich and Smith, 1973; Chamberlain, 1977), and activity measured there reflects contributions from all fiber subtypes but should be dominated by high-SR neuron activity, by virtue of the more frequent spiking of these fibers. It also reflects activity from an extended range of cochlear locations, within and outside the region most affected by the noise band we used.

Here, recordings obtained acutely revealed spontaneous activity reductions of greater than 50%. By 2 and 4 weeks, this activity had returned to control levels. These results suggest significant declines in the activity of high-SR neurons in the acute post-exposure time frame, with good recovery evident in chronic ears. Such recordings provide little direct information concerning the functional integrity of other fiber subtypes at this acute time point, as their spontaneous activity is not well-represented in the round window recorded signal. The sound-driven PSTRs are not helpful in disambiguating relative involvements at 24 h post-exposure, due to the effects of the noise on hair cells. At 2 and 4 weeks after noise, however, OHC-based thresholds and suprathreshold response levels are well recovered at all frequencies tested. The PSTR onset peaks, reflecting high-SR-dominated activity, are also at control levels, whereas the PSTR plateau, which captures low-SR activity, and the derived peak-to-plateau ratio are persistently and significantly altered. Together, results suggest good functional recovery of high-SR, but not low-SR neurons. Persistent loss of IHC synapses in the damaged region supports this hypothesis.

### Noise-Induced Cochlear Synaptopathy: Mice and Gerbils

The phenomenon of noise-induced and/or age-related primary cochlear neural degeneration has been documented in mice (Kujawa and Liberman, 2009; Sergeyenko et al., 2013), guinea pigs (Lin et al., 2011; Furman et al., 2013), chinchillas (Hickox et al., 2017; Hickman et al., 2018), gerbils (Gleich et al., 2016), rats (Lee et al., 2021) and rhesus macaques (Valero et al., 2017), as well as in humans (Wu et al., 2019, 2021). However, species and even strain differences in the permanence of noise-induced losses have been reported (Shi et al., 2013; Wang et al., 2015; Kaur et al., 2019; Hickman et al., 2020).

In the mouse, persistent CAP amplitude declines after noise have been highly correlated with persistent loss of synapses (Fernandez et al., 2020), whereas post-exposure recovery of neural function, as reflected in the recovery of CAP or ABR wave 1 amplitude toward control levels, has been observed when synapse counts return toward control levels with time and/or treatment (Fernandez et al., 2015, 2020, 2021; Suzuki et al., 2016; Kaur et al., 2019). In the current gerbil series, amplitudes of the whole nerve CAP recovered more completely than did synapse counts in the same frequency region. Recovery, in turn, must be shaped by characteristics of the exposure and vulnerabilities of the individual. As in most studies, a single exposure level is reported here, and exposure differences of only a few dB can have dramatic effects on the nature and degree of acute and chronic noise-induced injury (Kujawa, 2016; Kujawa and Liberman, 2019). Further clarification is needed on this key issue.

The auditory nerve CAP best reflects the activity of ANFs with robust, aligned onset responses, which should apply to both mouse and gerbil. In the final analysis, recovery differences may argue that ANFs of multiple SR subtypes, some with greater vulnerability and/or better recovery profiles, may have been acutely injured by exposure, as implied by the PSTR measures discussed above. We turn to single ANF recordings in these two species for additional insight.

In the CBA/CaJ mouse, the SR-based distribution of fibers appears homogenous along the cochlea, with around 10% of the units below 0.5 spike/s and around 50% above 20 spikes/s (see Taberner and Liberman, 2005, Figures 4, 5). In contrast, gerbils show a strong SR distribution gradient, with low-SR fibers accounting for about 9% of ANFs below 3.6 kHz and about 25% above 3.6 kHz. High-SR fiber proportions in these regions are ~75% and ~35%, respectively (see Figure 1C in Huet et al., 2016; Figure 5B in Huet et al., 2019). The larger proportion of low-SR fibers in the basal part of the gerbil cochlea, along with the weak contribution of this fiber pool to the CAP (Bourien et al., 2014), may help explain the recovery of the CAP to nearly normal amplitudes despite a ~30% synaptic loss. A similar amount of synaptic loss in mice (i.e., 30%) may include medium and/or high-SR fibers (only ~10% are low-SR), reducing CAP amplitudes. This SR-based difference between species may be an interesting working hypothesis to reconcile puzzling/inconsistent observations in studies of noise induced-hearing loss across species, including humans. In this framework, the use of the PSTR may prove informative as a tool to probe the auditory nerve in etiologies with synaptic and neural compromise.

## Data Availability Statement

The raw data supporting the conclusions of this article will be made available by the authors, without undue reservation.

## Ethics Statement

The animal study was reviewed and approved by the Institutional Animal Care and Use Committee of the Massachusetts Eye and Ear.

## Author Contributions

PJ performed the physiology recordings and assessed the cochlear tissues. PJ and AD contributed to the data analysis. JB designed the PSTR assays and analyzed the data. PJ, AD, JB, J-LP, and SK contributed to the design of experiments, writing and revising the manuscript. All authors contributed to the article and approved the submitted version.

## Conflict of Interest

The authors declare that the research was conducted in the absence of any commercial or financial relationships that could be construed as a potential conflict of interest.

## Publisher’s Note

All claims expressed in this article are solely those of the authors and do not necessarily represent those of their affiliated organizations, or those of the publisher, the editors and the reviewers. Any product that may be evaluated in this article, or claim that may be made by its manufacturer, is not guaranteed or endorsed by the publisher.
